# Deep Learning Based Air-Writing Recognition with the Choice of Proper Interpolation Technique

**DOI:** 10.3390/s21248407

**Published:** 2021-12-16

**Authors:** Fuad Al Abir, Md. Al Siam, Abu Sayeed, Md. Al Mehedi Hasan, Jungpil Shin

**Affiliations:** 1 Department of Computer Science & Engineering, Rajshahi University of Engineering & Technology, Rajshahi 6204, Bangladesh; 1603021@student.ruet.ac.bd (F.A.A.); 1603008@student.ruet.ac.bd (M.A.S.); abusayeed@cse.ruet.ac.bd (A.S.); mehedi@u-aizu.ac.jp (M.A.M.H.); 2 School of Computer Science and Engineering, The University of Aizu, Aizuwakamatsu 965-8580, Japan

**Keywords:** air-writing recognition, interpolation, time-series data, human–computer interaction, convolutional neural network

## Abstract

The act of writing letters or words in free space with body movements is known as air-writing. Air-writing recognition is a special case of gesture recognition in which gestures correspond to characters and digits written in the air. Air-writing, unlike general gestures, does not require the memorization of predefined special gesture patterns. Rather, it is sensitive to the subject and language of interest. Traditional air-writing requires an extra device containing sensor(s), while the wide adoption of smart-bands eliminates the requirement of the extra device. Therefore, air-writing recognition systems are becoming more flexible day by day. However, the variability of signal duration is a key problem in developing an air-writing recognition model. Inconsistent signal duration is obvious due to the nature of the writing and data-recording process. To make the signals consistent in length, researchers attempted various strategies including padding and truncating, but these procedures result in significant data loss. Interpolation is a statistical technique that can be employed for time-series signals to ensure minimum data loss. In this paper, we extensively investigated different interpolation techniques on seven publicly available air-writing datasets and developed a method to recognize air-written characters using a 2D-CNN model. In both user-dependent and user-independent principles, our method outperformed all the state-of-the-art methods by a clear margin for all datasets.

## 1. Introduction

In the last decade, we have grown accustomed to interacting with the digital world in various ways. Touchscreens and other electronic devices are common means for people to connect to the internet. The use of smartphones and other physical devices imposes the additional burden of transporting them and taking them out of one’s pocket to interact with them. The primary goal of next-generation technologies is to eliminate the necessity for intermediary physical devices, for instance, smartphones [1]. Virtual and augmented reality appears to be leading the way for the next generation of such technology, with output being projected directly into the eyes of the user(s) via specialized glasses [2]. Speech recognition is a well-studied approach that is thought to be a natural and intuitive way of interacting with technologies. However, speech recognition does not fulfill all parameters needed to communicate with technologies [1]. Gesture recognition is a method that has received a lot of interest in recent years which can be a communication method for next-generation technologies [2]. New technologies based on cameras, acceleration sensors, photosensors, and electromagnetic and auditory signals are emerging as new mediums of interaction employing gestures as an alternative to standard keyboards, touchpads, or other pushing and touching instruments [3]. In particular, for writing methods, traditional writing in touch sensors does not fit into Virtual Reality (VR), Augmented Reality (AR), and gesture-based technologies. To meet the requirement of touchless writing methods of next-generation technologies, air-writing is a promising solution. Air-writing is known as the act of writing letters or words with fingers or hand movements in free space [4]. It can be regarded as a special case of gesture, while the gesture is performing any kind of predefined movement in the air.

However, the recognition of air-writing characters is not a simple task [4]. Characters are distinguished from generic gestures by their fine-grained movement and they can be written in various ways by different people. In conventional writing, alphabets and numbers are written in a multi-stroke way in pen-and-paper-like systems [5]. Air-writing is different from the conventional writing system. Movements caused by lifting a pen are significantly less noticeable while writing in the air. Since users do not have the sense of touching anything, such as a pen or a piece of paper, they cannot see what is being written as they could see while writing on a piece of paper. As a result, the users may lose their sense of writing orientation in the space [2]. Despite all these difficulties, the recent advancements in the domain are promising.

In recent years, smartphones with in-built sensors have become widely available. Sensor data can be collected and preprocessed as per user requirement by creating a mobile application for the data collection process [2]. Hence, the domain of sensor datasets generated by smartphones is becoming larger day by day. Generally, while collecting the sensor data for gesture recognition, a user makes a gesture while holding or wearing motion sensors such as a gyroscope and/or an accelerometer [2,5,6]. On the other hand, the main challenge of building an air-writing recognition model using sensor data is the variability of signal length. Due to the nature of writing and data-recording procedures, variable signal length is evident [2], whereas deep learning methods such as the convolutional neural network require fixed-length signals in the training and prediction process. Fixed-length padding and truncation is a widely used process that pads or discards values from the start and/or end of a signal [7]. It is a simple method to implement but results in huge data loss, and therefore, the model is unable to capture significant features. On the contrary, interpolation is a statistical technique that predicts unknown values based on known values [8]. It ensures the fixed length of the signals by mapping the signal data into a predefined fixed length as a whole without discarding any portion of the signal. Therefore, data loss is minimal. Multiple interpolation techniques are available and well studied in the domain of image processing [9,10]. We investigated those various interpolation techniques on time-series data to obtain the fixed-length signals, i.e., employed interpolation methods on one-dimensional data instead of two-dimensional data. Furthermore, a well-structured, fine-tuned deep learning model can ease the training process and yield better accuracy in prediction. We designed a 2D-CNN model following the best practices proved in the literature and obtained state-of-the-art accuracy on seven publicly available datasets.

The organization of the rest of this paper is presented as follows: Section 2 presents the related works. Section 3 describes the dataset and the recognition methodology. In Section 4, the experimental analysis and evaluation of the methods are reported. Section 5 summarizes the paper emphasizing our contributions.

## 2. Related Work

Several researchers have recommended using motion sensors for gesture detection in recent years [6,11]. Liu et al. used an accelerometer signal which was captured from a Wii remote and recognized a predefined set of eight gestures using a DTW-based method [6]. The DTW-based method is a well-studied approach to deal with time-series data and air-writing recognition [12,13,14]. Chen et al. determined air-writing to be better than virtual keyboards in typing accuracy [4,15]. The authors also looked into identifying the beginning and end of each letter by the segments of the writing signal in a continuous data stream. A Wii remote was also used by Xu and Xue, where the users were given instructions about the order of movement for each of the air-written letters [16]. Li et al. used mobile-phone-captured motion signals performed by users and an LSTM-based deep neural network architecture to differentiate between twelve different handwritten characters consisting of six uppercase letters and six digits [17].

Since users perform air-writing by hand, the signals received from palm-worn devices may lead it to be harder to recognize the activities [2]. However, several studies have depicted that it is possible to classify the gestures from palm-worn devices [18,19]. Amma et al. recognized air-written letters with high accuracy using motion sensors positioned on the palm [20]. Xu et al. recognized textual input from wrist sensor data obtaining an accuracy of 98% [21]. Lin et al. investigated the orientations of the surfaces in which users were to write characters, the stabilization (support) point of the hand, and the rotation injection technique for data augmentation which uses a rotation matrix [22]. They obtained a remarkably high accuracy of 99.99% to recognize 62 characters by 10 subjects with a machine-learning-based approach. Chen et al. investigated real-time fingertip detection in frames captured from smart glasses. They built a synthetic dataset using Unity3D and proposed a modified mask regional convolutional neural network. Their method could detect fingertip for air-writing in a minimal length of time for each frame [23]. Kim et al. experimented with the WiTA dataset, which contains air-writing data for Korean and English alphabets collected by RGB cameras [24]. Bastas et al. experimented with handwritten digits, ranging from 0 to 9, which were structured as a multidimensional time-series data obtained via a Leap Motion Controller (LMC) sensor [25]. Tsai et al. suggested a reverse time-ordered algorithm to efficiently filter out unnecessary lifting strokes while writing in the air. To overcome the problem of different writing styles of different users, a tiered arrangement structure was presented by sampling the air-writing results with varied sample rates [26]. Arsalan et al. suggested an air-writing system based on a network of sparse radars and a 1D DCNN-LSTM-1D transposed DCNN architecture that can rebuild and identify the drawn character [27].

Moazen et al. attempted to recognize air-writing with a dataset containing 100 sets of samples of all 26 English letters collected from a single subject [28]. Uysal et al. proposed RF-Wri, a device-free machine-learning-based air-writing recognition framework that can differentiate 26 capital letters [29]. Yanay et al. allowed the users to write with their hands in the air naturally while capturing the motion signals by smart-bands [2]. In this experiment, the accelerometer and gyroscope signals were collected from the smart-bands to create a dataset of 15 sets of English alphabet for 55 subjects each. Finally, an average accuracy of 83.20% with the user-independent method and 89.20% with the user-dependent method was obtained in their experiment. To extract air-writing trajectories captured by a single web camera, Hsieh et al. proposed a hand-tracking algorithm [30]. Alam et al. experimented with a trajectory-based air-writing system where a depth camera was used which could track the fingertip to collect three-dimensional (3D) trajectories. They collected 21,000 trajectories and developed LSTM, CNN, and nearest-neighbor-based approaches and managed to obtain 99.17% accuracy [5]. Alam et al. proposed a trajectory-based air-writing character recognition system called CNN-LSTM, which used a combination of convolutional neural networks (CNNs) and long short-term memory (LSTM) to achieve 99.63 percent and 98.74 percent accuracy in the RTD and RTC datasets, respectively [31]. Alam et al. developed a technique for a finger-joint tracking-based character recognition system that uses 3D information to monitor the finger-joint and use the distance between the thumb tip and another finger-joint to identify a numerical digit, alphabet, character, special key, or symbol. Firstly, a single-hand-based digit recognition system was presented, which could be utilized with either the left or right hand. Secondly, a two-handed writing method was demonstrated, in which both hands were engaged at the same time. They achieved an overall accuracy of 91.95% for single-hand recognition and 91.85% for double-hand recognition, respectively [32]. In the absence of paired inertial and trajectory data, Xu et al. suggested an Air-Writing Translator model for learning bi-directional translation between trajectory and inertial domains. The researchers tested the suggested model on two publicly available datasets, 6DMG (in-air handwriting dataset) and CT (handwritten trajectory dataset), and showed that the model can reliably translate between the inertial and trajectory domains [33].

Our study differs from that of the existing studies in multiple aspects: (1) Though interpolation techniques are well studied in the digital image domain, they are often overlooked for time-series data [34]. In this paper, we experimented with various interpolation techniques on different publicly available air-writing datasets [2,5,35,36] and yielded the best interpolation technique for air-writing time-series data. (2) Proposing a well-structured, tuned deep learning model is necessary to gain the most of the data. We proposed a 2D-CNN model following the best practices proved in the literature [37,38,39]. These measures result in state-of-the-art performance, outperforming all the existing methods in both user-dependent and user-independent training principles by a clear margin for each of the seven air-writing datasets.

## 3. Materials and Methods

### 3.1. Dataset Description

We used a total of seven publicly available air-writing datasets in this research [2,5,35,36]. All of the datasets are different in the number of classes, the number of users or subjects (interchangeably used throughout the paper), the number of features, and the data acquisition method. Among the datasets, all of them were experimented with a user-dependent training principle and five of them were experimented with a user-independent training principle. In the user-dependent principle, samples from all users were considered for training and cross validation was employed for testing purposes. This is also known as the user-mixed principle [33]. Meanwhile, the user-independent principle overcame the necessity of the user registration process prior to testing. Here, one user was held off from testing while the rest of the users’ data were used for training. In Table 1, we present the summary of the datasets used in this study.

#### 3.1.1. Smart-Band Dataset

The smart-band dataset created by Yanay et al. contains air-writing data collected from 55 subjects [2]. Each subject wrote 15 sets of all 26 letters in the English alphabet in the air wearing a smart-band on their wrist. So, the dataset contains 390 samples of air-written letters per subject and there are 21,450 samples in total in the dataset.

The subjects wore the ‘Microsoft Band 2’ smart-band. The smart-band motion sensors, e.g., accelerometer and gyroscope, were used to record motion measurements in each sample. Both the accelerometer and gyroscope data were taken from three different axes (X, Y, and Z) with a maximum sampling rate of 62 Hz. To collect the signals from the motion sensors, an android mobile application was developed which ran on a Samsung Galaxy S8 smartphone. The smart-band was worn in the wrist of the hand with which the subject normally wrote, and the smartphone was held with another hand. The smartphone was connected to the smart-band via Bluetooth to collect data. Among the 55 subjects, 28 were females and the rest were males, while 46 subjects were right-handed, and the remaining 9 subjects were left-handed.

#### 3.1.2. Six-Dimensional Motion Gesture (6DMG) Datasets

The 6DMG dataset is a collection of alphanumeric air-writing characters, numerics, and gestures [35]. It was gathered using a hybrid framework in which an inertial sensor recorded tri-axial acceleration and tri-axial angular velocity, and an optical tracking device recorded the spatial coordinate trajectory. As a result, the 6DMG dataset includes both inertial and trajectory domain information. There is a total of 62 characters in this dataset, with 26 uppercase letters, 26 lowercase letters, and 10 numerals. The uppercase letters’ data were collected from 25 subjects, whereas only 6 subjects were used to compile data for the digit and lowercase dataset. Overall, the dataset comprises a total of 8570 samples, with 600 numeric samples, 6500 uppercase letter samples, and 1470 lowercase letter samples.

#### 3.1.3. RealSense-Based 3D Trajectory Digit and Character (RTD-RTC) Datasets

RealSense-based 3D Trajectory Digit and Character Datasets, abbreviated as RTD and RTC, respectively, are publicly available air-writing datasets containing the sequence of trajectory captured while writing English alphabets or digits in the free space. Both of the datasets were collected by Alam et al. [5,36]. In the RTC dataset, users wrote the English alphabets in front of sensor devices. The sensor data were gathered as a trajectory sequence. The fingertip was considered a substitute for a pen in the conventional pen–paper writing method. Users could write a character in front of an Intel RealSense SR300 camera, and the camera recognized the fingertip and collected it as a trajectory sequence. The direction of writing was a little different from the conventional multistroke style of writing, and it was written in a unistroke style [36]. In the RTD dataset, users wrote digits in front of the same device settings as RTC. The digits were written by the users in a defined manner [5].

Both the RTD and RTC datasets contain data as a sequence of trajectory, where each tuple indicates a trajectory. In each of the tuples, there are values of X-coordinate, Y-coordinate and Z-coordinate in the 3D Cartesian coordinate system, which was taken from the sensor device. However, in our experiment, we found that considering all three of the coordinate values from the RTD dataset produced meaningless output. Rather, only considering the X and Y coordinates made sensible outputs for the dataset. Therefore, the number of features for RTD and RTC dataset is 2 and 3, respectively, (see Table 1).

### 3.2. Data Preprocessing

In this work, we experimented with various interpolation methods while maintaining a fixed signal length of the air-writing sensor data. As the dataset is well balanced across the letters and free from missing values, no further data processing measures were necessary.

#### 3.2.1. Optimal Signal Length Selection

To feed the data into deep learning architectures such as the convolutional neural network for training and prediction, maintenance of a fixed length is a must. Due to the nature of the data acquisition procedure, the length of the signals is likely to differ. As we used interpolation techniques described in the previous section (see Section 3.2.2), we had to find a suitable length to shape all the signals. We applied two following techniques to obtain this goal:

1. We could consider the mean of the signal lengths such that the fixed length nearly split the data in half. Half of the signal length was less than the fixed length, so we had to upsample the data to increase the length. We downsampled the other half of the signals where the length of the signals was greater than the fixed length.
2. As loss occurs in downsampling the data, we could consider upsampling the maximum number of signals so that the data loss was kept to a minimum and the signal length was manageable.

#### 3.2.2. Fixed-Length Signals Using Interpolation Techniques

Interpolation is a statistical technique to predict probable unknown values based on known values [8]. It is widely used in image processing for reshaping images without the loss of the quality of the image or the visual experience and for improving the quality of the image. We used these interpolation techniques to obtain fixed-length signals to feed into the deep learning model without aggressive data loss.

**Figure 1:**
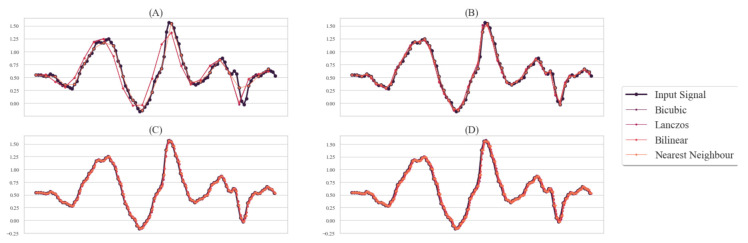
Different interpolation techniques applied on time-series data for upsampling and downsampling. (**A**) for 1/4 downsampling (signal length from 100 to 25), (**B**) for 1/2 downsampling (signal length from 100 to 50), (**C**) for 2× upsampling (signal length from 100 to 200), (**D**) for 4× upsampling (signal length from 100 to 400). The input signal was taken from the smart-band dataset.

In recent years, image processing methods with interpolation have gained importance for the capability of improving bad resolution images preserving the characteristics of the image [9]. The quality of the processed image is dependent on the chosen interpolation algorithm. There are different types of interpolation algorithms that have previously been developed. Among those algorithms, nearest neighbor, Bilinear, Bicubic, and Lanczos interpolation methods are widely used in different fields [10]. In Figure 1, we show the effects of different interpolation methods for upsampling and downsampling time-series signals. The interpolation techniques used in this paper are described below.

A Bicubic Interpolation: The Bicubic interpolation is the advanced version of cubic interpolation in a two-dimensional regular grid. The interpolation surface obtained here was smooth. Polynomial, cubic, or cubic convolution algorithm was used here. The cubic convolution determines the gray level value using the 16 closest pixels to the specified input coordinates and assigns the value to the output coordinates. The Bicubic interpolation kernel, W(x) [40] is defined as follows,
  (1) $$W\left(x\right)=\left\{\begin{array}{cc} {\left(a+2\right)|x|}^{3}-\left(a+3\right)x^{2}+1 & for\;|x|\;\leq 1\\ {a|x|}^{3}-{5a|x|}^{2}+8a\left|x\right|-4a & for\;1<|x|\;<2\\ 0 & otherwise \end{array}\right.$$
  where *a* is generally −0.50 or −0.75.
B Lanczos Interpolation: To smoothly interpolate the value of a digital signal between samples, the Lanczos filter is employed. Here, each sample of the given signal was mapped to a translated and scaled copy of the Lanczos kernel, L(x). The Lanczos kernel is a normalized sinc function which is windowed by a sinc window. The sinc window used is defined as the central lobe of a horizontally stretched sinc function sinc(x/a) for −a≤x≤a.
  (2) $$L\left(x\right)=\left\{\begin{array}{cc} sinc\left(x\right)sinc\left(\frac{x}{a}\right) & if\;-a<x<a\\ 0 & otherwise \end{array}\right.$$
  Equivalently,
  (3) $$L\left(x\right)=\left\{\begin{array}{cc} 1 & if\;x=0\\ \frac{asin\left(\pi x\right)sin\left(\frac{\pi x}{a}\right)}{\pi^{2}x^{2}} & if\;-a\leq x<a\;and\;x\neq 0\\ 0 & otherwise \end{array}\right.$$
  where *a* is a positive integer determining the size of the kernel, generally 2 or 3. The Lanczos kernel contains 2a−1 lobes. Among them, the number of positive lobes at the center is *a* and the other a−1 lobes are situated at each side which are alternating negative and positive lobes. For a one-dimensional signal with samples $s_{i}$, the value interpolated at an arbitrary real argument *x*, S(x) is obtained by the discrete convolution of those samples with the Lanczos kernel,
  (4) $$\sum_{i=\lfloor x\rfloor -a+1}^{\lfloor x\rfloor +a}s_{i}L\left(x-i\right)$$
  where the filter size parameter is defined as *a*. The sum is bounded in such a way that the kernel is 0 outside of the boundary [41].
C Bilinear Interpolation: The use of linear polynomials to generate new data points within the range of a discrete set of known data points is known as linear interpolation. Bilinear interpolation is accomplished by first performing linear interpolation in one direction and then repeating the process from the opposite direction. In bilinear interpolation, a value for a random position is determined by the weighted average of the four closest values. In the sense of image processing, the four closest values can be regarded as the four closest coordinates to the specified coordinate for which the value is to be determined. In this method, two linear interpolations are performed. One linear interpolation is performed in a direction and the next is performed in the perpendicular direction. The output is smoother than the original input value set. When all distances between the data points are equal, then the interpolated value is their sum divided by four. Here, the interpolation kernel, H(x) is
  (5) $$H\left(x\right)=\left\{\begin{array}{cc} 0 & if\;|x|\;>1\\ 1-|x| & if\;|x|\;<1 \end{array}\right.$$
  where *x* is the distance between two points to be interpolated.
D Nearest Neighbor Interpolation: Nearest neighbor interpolation is the most simple interpolation technique [42,43]. In this method, each interpolated output value is generated with the closest sample point in the input. This method produces discontinuous interpolated data [44]. The interpolated point $X_{i}$ is determined by
  (6) $$X_{i}=\left\{\begin{array}{cc} X_{B} & if\;i<\frac{a+b}{2}\\ X_{A} & if\;i\geq \frac{a+b}{2} \end{array}\right.$$
  where *a* and *b* are the indexes of $x_{A}$ and $x_{B}$ and a<i<b.

### 3.3. Convolutional Neural Network Architecture

A convolutional neural network, abbreviated as CNN, is a type of deep neural network for processing raw visual data, inspired by the organization of the visual cortex of animals [45,46] and made to learn spatial hierarchies of features, from low-level to high-level patterns, automatically and adaptively using convolutional and pooling layers and activation functions. CNNs are widely employed in computer vision tasks. Classification, object localization and detection, segmentation, and pose estimation are some of these tasks, to name a few. Lately, they have gained popularity in the research area of human activity recognition [47,48,49]. They have also been used for the classification of time-series data obtained from accelerometers, gyroscopes, and other sensors [50,51,52,53]. We proposed a convolutional neural network following the best practices that adapts well for air-writing recognition utilizing time-series data from various sensors.

Our proposed convolutional neural network architecture was composed of four groups of layers other than the input layer, where the first three groups consisted of a couple of two-dimensional convolution, maxpooling, and dropout layers for feature extraction. We flattened the output from the third convolutional group, and a dense layer accompanying dropout was employed with the softmax activation function to gain the prediction. Except for the prediction layer, we used Rectified Linear Units (ReLUs) as the activation function throughout the network.

The input of the network was the tensor of format: l×f×1, where *l* is the signal length, *f* is the number of features (time-series signals) in the dataset. This tensor was therefore propagated through the convolutional layers. Each of the convolutional groups was constructed using conv-conv-maxpool-dropout layers, sequentially. The core objective for consecutive convolutional layers without pooling layers is to replace a single layer with a larger receptive field rather than skipping any pooling. It is a widely used construct for developing convolutional neural network [37,54,55,56]. We incorporated two non-linear convolutional layers instead of a single one with a larger filter size to make the decision function more discriminative. Additionally, this approach decreased the number of trainable parameters [37].

Dropout has been an integral part of deep neural networks since their inception [57]. Wu and Gu studied the effects of dropout on different layers of CNNs and showed that the dropout of maxpooling and fully-connected layers performed best [38]. Therefore, we used dropout after every maxpooling layer and in the fully-connected layer where the percentage, *p* values of the dropouts were chosen according to the suggestions given by Park and Kwak [39]. The network architecture specification is provided in Table 2, considering the smart-band dataset.

### 3.4. Experimental Settings and Evaluation Metrics

To accelerate our training procedure, NVIDIA Tesla T4 GPU and 12 GB of RAM were used provided by Google Colaboratory free of charge [58]. We used OpenCV library [59] to interpolate the time-series data and Keras API over TensorFlow backend [60] to create the CNN model.

We measured the performance of the model by recognition accuracy (see Equation (7)) in user-dependent and user-independent principles. In the user-dependent principle, 10-fold cross-validation accuracy was reported for smart-band, RTC, and RTD datasets, and 5-fold cross-validation accuracy was reported for the variations of the 6DMG dataset to compare our method with the state-of-the-art methods. The evaluation metric was considered for multiclass classification, as we had a various number of digits and characters in different datasets (see Table 1). Note that the datasets are mostly balanced regarding the number of samples per letter per subject where applicable. Therefore, the evaluation of classification performance using accuracy alone was justified.

(7) $$Accuracy=\frac{Number\;of\;correct\;predictions}{Total\;number\;of\;predictions}$$

## 4. Experimental Analysis

In this section, we experimented extensively with the various interpolation techniques to find the best method among them and a suitable fixed signal length, *l* for interpolation using the smart-band dataset developed by Yanay et al. [2]. We performed comparative analysis with other existing signal length unification methods, such as padding and truncation, with the best interpolation method. Finally, we employed our methodology on six other air-writing datasets [5,35,36] to verify our findings and yielded state-of-the-art performances.

### 4.1. Searching Optimal Signal Length

**Figure 2:**
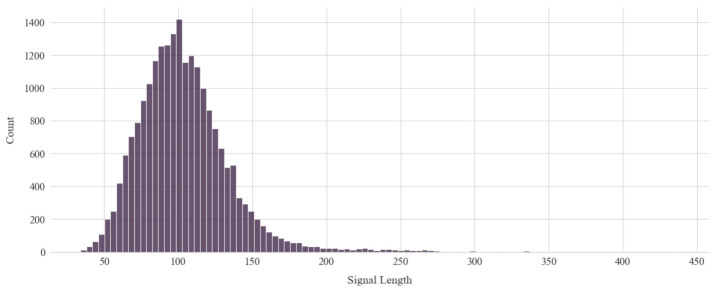
Histogram of the length of all samples from smart-band dataset.

The process of recording the air-writing data results in different signal lengths. In Figure 2, we show a histogram of the lengths of all the samples from the smart-band dataset. Along with the interpolation methods, the fixed length of the signals plays an important role in data preprocessing, as we required a unified length to fit data into the 2D-CNN model.

Denoting the fixed signal length using *l*, we would have a matrix of size l×6 for each sample of the letters as we had six time-series data for each sample (see Section 3.1.1). Now, from the length distribution of each sample (see Figure 2), we can see that it is itself a matter of choice to select the fixed signal length (see Section 3.2.1). From the statistical characteristics, we considered the two following aspects. Firstly, we considered a round mean of the length distribution where approximately half of the data would be downsampled and half would be upsampled. However, from Section 3.2.2 and Figure 1, we can see that each interpolation method densely populates the time-series while upsampling, but for downsampling, we may significantly lose important data features. Therefore, secondly, if we upsample the majority of the data, we may have some data redundancy, but data loss is mostly prevented. Therefore, we considered 100 and 200 to be the signal length in our experimentation, where 100 is used to balance interpolation for upsampling and downsampling and 200 for upsampling the majority of data so that the data loss is minimized. Hence, the shape of the matrix containing all of the samples will be 21,450 × l × 6, where l=100,200.

### 4.2. Effects of Various Interpolation Techniques

For the signal length, l=100, we considered different methods for upsampling and downsampling as approximately half of the data was upsampled and half of the data was downsampled. Therefore, we had 20 different combinations of interpolation methods (see Table 3), whereas, for length l=200, considering 20 different combinations did not make any sense, as there are very little data left to be downsampled. So, we considered the same interpolation method for both upsampling and downsampling. To minimize the time taken to fine-tune the model, five random users (user no. = {22, 10, 4, 47, 40}) were taken to build the test set and the rest of the users remained in the training set. For different interpolation methods and fixed signal lengths, the performance of the classification model is presented in Table 3.

From Table 3, we can see that, using signal length, l=100, for all combinations of the interpolation methods, the results are marginally poor as we lose important temporal features for downsampling time-series data. For signal length, l=200, Bicubic interpolation for both the up and downsampling performs best, though the method did not yield data much compared to the other methods. As we upsampled most of the data for l=200, temporal data loss was minimal. Therefore, we had to select the optimal fixed signal length so that most of the data were upsampled. Consideration of the computation trade-off is critical as the signal length should not be too large to result in data redundancy.

In a similar setting, we performed comparative analysis with the other existing signal length unification methods such as pre-sequence padding and truncation and post-sequence padding and truncation [7]. For a set of variable length signals, finding a unified length for all the signals may result in both padding (if the length of a particular signal is less than the fixed signal length) and truncation (if the length of a particular signal is greater than the fixed signal length). Here, the idea of padding and truncation is kind of similar to upsampling and downsampling, respectively. The sampling rate of the signal is changed accordingly for up and downsampling and the overall characteristics of the signal are kept where padding fills the extra slots by zeros and truncation cuts the signal to make the signal as required. The words, pre and post of the methods refer to the part of the sequence where the padding by zero or the truncation takes place.

In Table 4, it is shown that for a wide range of sequence lengths, the padding and truncation methods performed worse than the Bicubic interpolation technique. As the signal length increased to 400, the inference time of the model and the number of floating-point operations also increased, but the performance of the model decreased for both cases of the padding and truncation method. Meanwhile, the Bicubic interpolation techniques performed much better for l=200, which is also computationally optimal. We also evaluated Bicubic interpolation for l=50 and l=400, and the method performed surprisingly well for l=50. In this case, pre-sequence padding and truncation and post-sequence padding and truncation were 59.87% and 48.15% accurate, respectively, where Bicubic interpolation achieved 84.94% accuracy. For l=400, the accuracy of the model was reduced than that of l=200 due to data redundancy. Therefore, we selected l=200 as the optimal signal length for the smart-band dataset. Following the same procedure, we also selected the signal length, *l* for other datasets (see Table 5).

### 4.3. Results and Discussions

In the literature, air-writing recognition is evaluated under two different training principles [2,16,33]: (1) the user-dependent principle and (2) the user-independent principle. The definitions of the principles are stated in the dataset description section (see Section 3.1).

Our method outperformed all the state-of-the-art methods in both principles for all the datasets. We reported the performances of our method in comparison with the previous methods [2,4,5,16,31,33,36,61,62,63], shown in Table 6 and Table 7. From Table 6, compared with the existing methods employed on the smart-band, RTC, and RTD datasets, our proposed method achieved the best recognition performance in terms of accuracy in both user-dependent and independent principles by a clear margin. For the variations of the 6DMG dataset, the performance comparison is presented in Table 7. Our method outperformed all the existing methods for digits, lowercase letters, uppercase letters, and all of these combined for both training principles. Empirically, under the user-dependent principle, our model achieved 100% accuracy for digits, which was verified by multiple random splits in cross-validation. Furthermore, we achieved a 3.55% accuracy gain for all the classes combined and 0.52%, 1.62%, and 2.24% accuracy gain for digit, lowercase, and uppercase datasets, respectively, in the user-independent principle.

## 5. Conclusions

Air-writing recognition will be essential in the post fourth industrial revolution world. In this study, we developed a method to recognize characters and digits in the English alphabet using time-series data. Sensor data preparation for deep learning methods while ensuring minimal data loss is a challenging task. We extensively explored different interpolation techniques which are widely used for images but often overlooked for time-series signals. Our experiment shows that interpolating the raw data using the Bicubic interpolation algorithm provides the best results in our use case. Upon this interpolated data, we trained our proposed 2D-CNN model to classify the letters, which outperformed the state-of-the-art methods by a clear margin. Fine tuning to the recognition system will be necessary before the real-world deployment of the air-writing recognition system. Furthermore, we can hybridize user-independent and user-dependent methods, create a guidance or feedback loop to the user, and introduce an auto-correction mechanism. Additionally, we can explore the subjects’ characteristics extensively. Though the datasets particularly indicated some of the characteristics of the subjects, we did not consider those facts, as we intend to make the system more generalized. Last but not least, further research is essential to recognize words in similar settings, as it is much more challenging than recognition of an isolated character or digit. To build a fully functional air-writing recognition system, all of these issues have to be addressed. We present our work as one of the steppingstones in that path.

## Figures and Tables

**Table 1:** Summary of the datasets used in this study.

Dataset	No. of Classes, *n*	No. of Features, *n*	No. of Users	No. of Samples	Training Principle	
					User Dependent	User Independent
RTD * [5]	10	2	10	20,000	✓	✗
RTC * [36]	26	3	10	30,000	✓	✗
Smart-band [2]	26	6	55	21,450	✓	✓
6DMG-digit [35]	10	13	6	600	✓	✓
6DMG-lower [35]	26	13	6	1470	✓	✓
6DMG-upper [35]	26	13	25	6500	✓	✓
6DMG-all [35]	62	13	25	8570	✓	✓

* The authors did not disclose user data. Therefore, training in user-independent principle cannot be performed.

**Table 2:** Network architecture of the 2D-CNN model for air-writing recognition based on smart-band dataset.

Operation Group	Layer Name	Filter Size	No. of Filters	Stride Size	Padding Size	Activation Function	Output Size *	No. of Parameters *
-	Input	-	-	-	-	-	200×6×1	0
Group1	Conv1-1	2×2	32	1×1	1×1	ReLU	200×6×32	160
	Conv1-2	2×2	32	1×1	1×1	ReLU	200×6×32	4128
	MaxPool1	2×2	1	2×2	0	-	100×3×32	0
	Dropout	p=10%					100×3×32	0
Group2	Conv2-1	2×2	64	1×1	1×1	ReLU	100×3×64	8256
	Conv2-2	2×2	64	1×1	1×1	ReLU	100×3×64	16,448
	MaxPool2	2×2	1	2×2	0	-	50×2×64	0
	Dropout	p=20%					50×2×64	0
Group3	Conv3-1	2×2	128	1×1	1×1	ReLU	50×2×128	32,896
	Conv3-2	2×2	128	1×1	1×1	ReLU	50×2×128	65,664
	MaxPool3	2×2	1	2×2	0	-	25×1×128	0
	Dropout	p=20%					25×1×128	0
Group4	Flatten	-	-	-	-	-	3200	0
	Dense	-	-	-	-	ReLU	512	1,638,912
	Dropout	p=50%					512	0
	Dense	-	-	-	-	Softmax	26	13,338
							Total	1,779,802

* Output size and no. of parameters vary based on the number of features and signal length, *l*, depending upon the dataset under consideration. For smart-band dataset, the number of features is 6 and the signal length, *l* is 200 (see Table 1 and Section 3.2.1). Therefore, we yield this 2D-CNN network. The layers that construct the network and the attributes remain the same for all datasets.

**Table 3:** Interpolation methods in different upsampling and downsampling settings.

Upsampling Method	Downsampling Method	Signal Length, *l*	Accuracy	
			Avg. (%)	Std.
Bicubic	Bicubic	100	87.35	0.41
	Lanczos		87.21	0.59
	Bilinear		87.76	0.45
	Nearest neighbor		87.04	0.21
Lanczos	Bicubic	100	87.54	0.18
	Lanczos		86.50	0.70
	Bilinear		86.84	0.46
	Nearest neighbor		86.73	0.27
Bilinear	Bicubic	100	86.91	0.77
	Lanczos		86.77	0.63
	Bilinear		86.25	0.80
	Nearest neighbor		87.38	0.09
Nearest neighbor	Bicubic	100	87.38	0.37
	Lanczos		87.32	0.76
	Bilinear		86.67	0.58
	Nearest neighbor		87.08	1.24
Bicubic	Bicubic	200	88.54	0.31
Lanczos	Lanczos		87.35	0.31
Bilinear	Bilinear		88.46	0.19
Nearest neighbor	Nearest neighbor		88.08	0.59

**Table 4:** Comparative analysis of Bicubic interpolation with padding and truncation methods.

Approach	Sequence Length, *l*	# Padded or Upsampled Samples	# Truncated or Downsampled Samples	# Flops	Inference Time (ms)	Accuracy	
						Avg. (%)	Std.
Pre-sequence Padding and Truncation	50	212	21,238	599,177	1.648	59.87	0.52
	100	10,892	10,558	992,393	1.492	84.57	0.30
	200	21,161	289	1,778,825	1.709	86.58	0.37
	400	21,449	1	3,417,225	2.056	86.38	0.24
Post-sequence Padding and Truncation	50	212	21,238	599,177	1.429	48.15	0.44
	100	10,892	10,558	992,393	1.843	80.25	0.23
	200	21,161	289	1,778,825	1.770	85.84	0.42
	400	21,449	1	3,417,225	2.519	85.63	0.35
Bicubic Interpolation	50	212	21,238	599,177	1.475	84.98	0.35
	100	10,892	10,558	992,393	1.533	87.35	0.41
	200	21,161	289	1,778,825	1.687	88.54	0.31
	400	21,449	1	3,417,225	2.430	88.02	0.48

**Table 5:** Selected signal length, *l* for all datasets.

Dataset	Min	Max	Signal Length, *l*
RTD	18	150	125
RTC	21	173	125
Smart-band	34	438	200
6DMG-digit	29	218	175
6DMG-lower	27	163	150
6DMG-upper	27	412	250
6DMG-all	27	412	250

The terms “Min” and “Max” represent the minimum and maximum length of the signals in that particular dataset, respectively.

**Table 6:** Performance evaluation for user-dependent and independent method on smart-band and RealSense-based Trajectory datasets. Abbreviations of the approaches are given in the Abbreviations section of this paper.

Training Principle	Approach	Accuracy		
		Smart-Band	RTC	RTD
User-dependent principle (10-fold CV)	KNN-DTW based [2]	89.20	-	-
	2D-CNN based [36]	-	97.29	-
	LSTM based [5]	-	-	99.17
	CNN-LSTM fusion [31]	-	98.74	99.63
	Proposed	91.34	99.63	99.76
User-independent principle	1D-CNN [2]	83.20	-	-
	Proposed	85.59	-	-

**Table 7:** Performance evaluation for user-dependent and independent methods on the 6DMG dataset. Abbreviations of the approaches are given in the Abbreviations section of this paper.

Training Principle	Approach	Accuracy							
		Digit		Lower		Upper		All	
		Avg. (%)	Std.	Avg. (%)	Std.	Avg. (%)	Std.	Avg. (%)	Std.
User- dependent principle (5-fold CV)	HMM-based [4]	-	-	-	-	98.16	2.37	-	-
	LSTM-bases [61]	97.33	1.49	96.80	0.57	98.34	0.50	94.75	0.31
	CRF-CNN fusion [62]	-	-	-	-	98.57	-	-	-
	BiLSTM-CNN fusion [63]	99.33	-	-	-	99.27	-	-	-
	CHMM-based [16]	99.00	1.09	98.22	0.73	97.29	0.66	95.91	0.47
	UDA [33]	99.78	0.03	98.94	0.08	99.55	0.06	97.03	0.11
	Proposed	100.00	0.00	99.47	0.39	99.80	0.20	98.99	0.23
User- independent principle	CHMM [16]	96.70	4.08	76.38	5.25	91.03	1.54	62.69	2.91
	UDA [33]	98.74	0.34	92.86	0.48	96.99	0.45	87.69	0.58
	Proposed	99.26	0.12	94.48	0.45	99.23	0.94	91.24	0.86

## Data Availability

All the datasets used in this research are publicly available. Smart-band dataset is available at: http://bigdatalab.tau.ac.il/shared_resources/datasets/airwriting_dataset.zip, accessed on 15 November 2021. All the variations of 6DMG dataset can be accessed at https://mingyu623.github.io/6DMG.html, accessed on 15 November 2021, RTC dataset can be found at https://shahinur.com/en/rtc/, accessed on 15 November 2021, and RTD dataset is available at https://shahinur.com/en/rtd/, accessed on 15 November 2021.

## Abbreviations

The following abbreviations are used in this manuscript:

RTD	RealSense Trajectory Digit
RTC	RealSense Trajectory Character
6DMG	6-Dimentional Motion Gesture
CNN	Convolutional Neural Network
KNN	K-Nearest Neighbors
DTW	Dynamic-Time-Warping
UDA	Unsupervised Domain Adaptation
CRF	Conditional Random Fields
HMM	Hidden Markov Model
CHMM	Continuous Hidden Markov Model
LSTM	Long Short-term Memory
BiLSTM	Bidirectional Long Short-term Memory
